# From Tumor Immunosuppression to Eradication: Targeting Homing and Activity of Immune Effector Cells to Tumors

**DOI:** 10.1155/2011/439053

**Published:** 2011-12-08

**Authors:** Oana Draghiciu, Hans W. Nijman, Toos Daemen

**Affiliations:** ^1^Molecular Virology Section, Department of Medical Microbiology, University Medical Center Groningen (UMCG), University of Groningen, HPC EB88, P.O. Box 30.001, 9700 RB Groningen, The Netherlands; ^2^Department of Gynecology, University Medical Center Groningen, University of Groningen, 9700 RB Groningen, The Netherlands

## Abstract

Unraveling the mechanisms used by the immune system to fight cancer development is one of the most ambitious undertakings in immunology. Detailed knowledge regarding the mechanisms of induction of tolerance and immunosuppression within the tumor microenvironment will contribute to the development of highly effective tumor eradication strategies. Research within the last few decades has shed more light on the matter. This paper aims to give an overview on the current knowledge of the main tolerance and immunosuppression mechanisms elicited within the tumor microenvironment, with the focus on development of effective immunotherapeutic strategies to improve homing and activity of immune effector cells to tumors.

## 1. General Introduction

In the beginning of the 20th century, the concept according to which the immune system can be manipulated for tumor prevention or tumor treatment has emerged. Around half a century later, Burnet postulated the existence of a complex immunological mechanism capable of eliminating potentially malignant cells and, thus, gave birth to what would afterwards be called the cancer immunosurveillance theory [1]. In later years, strong evidence supporting the existence of intricate antitumor immune responses lead to the more exhaustive concept of cancer immunoediting. According to this concept, the multistep process of cancer development consists of three phases. The first phase, of elimination, is similar to the cancer immunosurveillance theory. Malignant cells, generated after genetic modifications that may occur during cell division cycles, present the singular property of expressing tumor antigens, a feature which makes them immunologically distinguishable from nonmalignant cells. Recognition of these tumor antigens by cells belonging to the host immune system leads to development of antitumor immune responses. Within the second phase, of equilibrium, a dynamic balance between the tumor microenvironment and the host immune responses is established. However, due to the negative activity of the tumor microenvironment as a dynamic inducer of immune cell anergy or death [2, 3], these antitumor immune responses are apparently insufficient to completely eradicate tumors. Hence, the third phase, of escape, consists of development of immune resistant tumor variants into fully grown and progressive clinical tumors [4, 5]. Here, the concept of cancer immunotherapy comes into play. Although the host immune system is clearly capable of recognizing cancer cells [6], the ability to which it can control tumor growth remains very limited. Different explanations can be envisaged to justify the decreased antitumor activity of the immune system. All of them take into account two major obstacles: on one hand, reduced homing of immune cells to the tumor site and, on the other, hampering of the antitumor immune functions due to tumor microenvironment or immunomodulatory properties of suppressive cell populations. Cancer-directed immunotherapies encompass diverse attempts either to stimulate the antitumor immune system or to inactivate and deplete protumor immune cell populations. Effective antitumor immunotherapeutic strategies take into account the complex interplay between innate, nonspecific and adaptive, antigen-specific, immune responses. 

This paper aims to give an overview on the current knowledge of the main tolerance and immunosuppression mechanisms elicited within the tumor microenvironment, with the focus on development of effective immunotherapeutic strategies to improve homing and activity of immune effector cells to tumors.

## 2. The Balance of Immune Surveillance in the Tumor: Navigating between Scylla and Charybdis

An increasing body of evidence substantiates the concept that specific cell populations from both the innate and adaptive immune systems interact with developing tumors and frequently contribute to the arrest of tumor growth and induce tumor regression in animal models and cancer patients. To counteract the antitumor activity of these effector cells, regulatory cell populations have emerged, capable of suppressing the antitumor immune responses through a large array of mechanisms. These silencing or suppression mechanisms can be functionally divided in two main categories: tolerance mechanisms, characterized by the absence of an immune response only to a specific set of antigens and maintenance of normal responses to all other antigens and immunosuppression mechanisms, characterized by an impaired ability of the immune system to fight cancer development.

### 2.1. Induction of Tolerance Mechanisms

Most often, tolerance mechanisms are directed against the antitumor activity elicited by cell populations belonging to the adaptive immune system. The main targets of these tolerance mechanisms are Th1 CD4^+^ T cells and cytotoxic CD8^+^ T lymphocytes (CTLs). Apart from these adaptive immune populations, dendritic cells (DCs) are a distinct cell subset with the capacity to initiate primary and secondary T-lymphocyte responses against developing cancer, thus representing a putative target for tolerance induction. Both the importance and relevance of these immune populations and the tolerance mechanisms they are the target of are shortly addressed below.

#### 2.1.1. Dendritic Cells

Alongside macrophages and B lymphocytes, DCs comprise one of the three main professional APC populations. Within the context of tumor development, their crucial importance stems from the capacity to engulf, process, and present tumor-associated antigens (TAAs) and thus generate tumor-specific immunity. Generation of potent antitumor immunity by DCs is the result of a complex process comprising three major steps: proper presentation of TAAs to T lymphocytes, activation of T lymphocytes in a specific manner as a response to TAAs presentation, and homing of these specific T cells to the tumor site, where they exert cytolytic activity against tumor cells expressing the TAAs [7]. Tumor escape mechanisms developed during cancer progression can occur at any of these various levels. With respect to the first step, these escape mechanisms generally translate into a deficit in antigen presentation. This deficit stems from two major sources: on one hand, a decreased number and function of APCs, and on the other, a semimature phenotype. One of the earlier studies indicating the effects of defective antigen, presentation by DCs is performed in a murine model bearing tumors transfected with a human p53 minigene. Both *in vitro* restimulation of T cells isolated from either control or tumor-bearing mice and *in vivo* induction of CTLs by DCs from tumor-bearing mice were significantly decreased in comparison with the same effects exerted by DCs isolated from control mice [8]. Later research in this direction further substantiates these findings in various clinical models. A study performed on DCs isolated from renal cell carcinoma patients indicates that less than 10% of the total DC population represents activated cells capable of antigen presentation and T cell stimulation [9]. The situation proves to be similar in patients with both advanced breast cancer [10, 11] and non-Hodgkin's lymphoma [12]. Moreover, DCs exposed to indoleamine 2,3-dioxygenase [13], transforming growth factor-beta (TGF-*β*) or prostaglandins, have been shown to induce tolerance and anergy leading to failure of recognizing tumor cells.

#### 2.1.2. Th1 CD4^**+**^ T lymphocytes

Although not directly capable of antitumor activity due to their lack of cytotoxic and phagocytic properties, CD4^+^ T cells, also known as mature T helper cells, play a crucial role in the initiation and activation of the antitumor immune response. In accordance with their phenotypic characteristics and function, CD4^+^ T cells can be divided in two types. Type 1, IL-12 polarized CD4^+^ T cells (Th1) provide help to cytotoxic CD8^+^ T cells, amongst others by stimulating their proliferation and inducing IFN-*γ* secretion once antigen-specific immunity has developed [14]. In contrast, type 2, IL-4 polarized [15] CD4^+^ T cells (Th2) secrete cytokines which induce neutralizing antibody production by B cells, thus directing immunity towards a tumor-promoting type 2 response.

#### 2.1.3. Cytotoxic CD8^**+**^ T lymphocytes

Cytotoxic T cells constitute a subgroup of T lymphocytes able to induce death of tumor cells and infected or otherwise dysfunctional somatic cells, following their activation. The activation process of cytotoxic CD8^+^ T lymphocytes relies on various simultaneous interactions between molecules which are expressed on the surface of the CD8^+^ T cell itself and corresponding molecules located on the surface of the antigen-presenting cell (APC). The first activation cue of CD8^+^ T cells consists of the interaction between their membrane T cell receptor (TCR) and peptide-bound MHC class I molecules located on the surface of APCs. Following this cue, a second signal comprising of interactions between the costimulatory molecules CD28 (located on the surface of CD8^+^ T cells) and CD80 or CD86 (located on the surface of APCs) can develop. Depending on the case, this second signal can be substituted by cytotoxic T cell stimulation with cytokines released by helper T cells. Similarly to CD4^+^ T cells, CD8^+^ T cells can also be divided in different subsets [16], according to their phenotypical and functional properties. Naïve CD44^low^ CD8^+^ T cells are differentiated mature T cells that have not yet encountered their cognate antigen in the periphery. Upon antigen recognition, they become memory CD44^high^ CD8^+^ T cells with a higher sensitivity to TCR/CD8 signaling in response to subsequent antigen stimulation [17]. Effector memory CD8^+^CD44^+^ T cells (Tem), characterized by low expression of markers necessary for cellular extravasation (e.g., CD62L), have been shown to restore systemic antitumor immunity in mouse models of lung and mammary carcinoma [18]. When compared with Tem cells, central memory T cells (Tcm), phenotypically defined as CD8^+^CD44^+^CD62L^+^CD127^+^, confer superior immunological protection against viruses, bacteria [19], and cancer [20]. antitumor effector T cells can be obtained by systemic delivery of IL-12 and GM-CSF to tumors or by activation of tumor-resident CD8^+^ T effector/memory cells [21]. By releasing various cytokines, such as perforin and granzyme B, these effector T cells are capable of inducing apoptotic death of tumor cells. The activity of these adaptive immune-cell populations is being continuously targeted by the tumor microenvironment, through a versatile array of either tolerance or immunosuppression mechanisms. 

#### 2.1.4. Tolerance Mechanisms

When talking about cancer development and progression, one should take into account two main types of alterations within the tumor environment: effector-cell related tolerance or immunosuppression and tumor-cell associated alterations. Intrinsic alterations of the tumor cells lead to decrease or disappearance of immunogenicity, whereas extrinsic alterations are induced by the tumor cells themselves, however exerting their activity on effector T cells within the tumor microenvironment. The latter of the two comprises more varied and versatile escape mechanisms, as they can either elicit a proximal effect, on the surrounding microenvironment or a distant effect, on the host immune system giving rise to the state of immunological tolerance. A proximal effect of colon cancer cells, which leads to evasion of FasL mediated cell death, is secretion of decoy receptors that bind and neutralize FasL [22]. On the other hand, distant effects exerted on the host immune system consist of a wide array of tolerance mechanisms. One very efficacious tolerance mechanism is deletion of effector T cells due to expression of death-inducing ligands by cancer cells [23, 24]. Direct tolerization of antitumor T cells by tumor cell-induced TGF-*β* signaling is another highly effective mechanism, leading to inhibition of master transcriptional regulators of CD4^+^ T cells [25] and significantly decreased function and frequency of CTLs (cytotoxic T lymphocytes) in a thymoma mouse model [26]. The main tolerance mechanisms leading to decreased numbers of antitumor effector T cells, coupled with increased numbers of low-affinity autoreactive T cells [27], are constituted by ignorance and anergy. Immunological ignorance is characterized by lack of contact with the antigens able to induce phenotypical changes, whereas anergy arises after negative regulation induced by different types of host factors (e.g., suppressor cells, their secreted cytokines) [28]. Other competent tolerance mechanisms are deficient priming of antitumor effector T cells [29] and increased expression of inhibitors which block complement mediated lysis of tumor cells [30]. However, regardless of the tolerance mechanism exerted by tumor cells, the end result consists of reduced or completely suppressed cytolytic activity of intratumoral effector T cells. Strategies aimed at increasing the activity of these immune effector cells at the tumor site will be addressed in Section 3.2 of this paper.

### 2.2. Induction of Immunosuppression Mechanisms

When compared to mechanisms of tolerance induction, the machinery of antitumor Immunosuppression is more versatile, since it encompasses a large variety of tools used by the tumor environment to target various mechanisms of inhibition of tumor growth and development. From a cellular point of view, the most widely encountered suppressive cell populations within the tumor environment are macrophages, myeloid derived suppressor cells, and regulatory T cells. The mechanisms by which these cell populations manage to give rise to tumor-immune escape are described below. 

#### 2.2.1. Tumor Associated Macrophages (TAMs)

Tumor-induced recruitment and expansion of regulatory cell populations is aimed at both the innate and adaptive immune systems. Concerning recruitment of suppressive innate immune populations, one clear example is given by TAMs. Similarly to CD4^+^ T cells in adaptive immunity, the innate immune populations of macrophages can be either anti- or protumorigenic, depending on their phenotype [31]. Antitumorigenic infiltrating macrophages, “classically activated” by the action of microenvironmental signals such as IFN-*γ* and bacterial factors, are polarized towards the M1 phenotype [32] and elicit cytotoxic activity against tumor cells *in vivo* [33], through their production of Th1 cytokines and iNOS. These macrophages also have the capacity to function as antigen presenting cells [34] that activate CTLs. On the other hand, TAMs are “alternatively” activated by Th2 cytokines such as IL-4 or IL-13 [35] towards an M2 non-cytotoxic phenotype. These M2 macrophages are frequently found in solid tumors, where they promote remodeling of the extracellular matrix and secrete growth factors, therefore, inducing tumor-specific neoangiogenesis [36]. Also, different studies have underlined their capacity to cause tumor growth both directly, by production of cytokines that stimulate proliferation of tumor cells [37], and indirectly by stimulating proliferation of endothelial cells [38]. For example, in the HPV16 E6- and E7-expressing TC-1 tumor mouse model, TAMs were shown to cause suppression of the antitumor T-cell response [39], while their secreted IL-10 cytokine subsequently induced a regulatory T cell phenotype [40].

#### 2.2.2. Myeloid-Derived Suppressor Cells (MDSCs)

MDSCs represent a highly heterogenic population of incompletely matured granulocytes, macrophages, and dendritic cells [41], with different morphology, functions, and differentiation conditions, when compared to TAMs [42]. Although MDSCs are capable of immune response regulation in healthy individuals, it has been observed that they dramatically expand during cancer development and treatment (Draghiciu O, Walczak M, Nijman HW, and Daemen T, unpublished observations), inflammation conditions or chronic infections [43, 44]. Characterized by a high phenotypical variety, they can be generally identified in mice as CD11b^+^Gr1^+^ cells [45]. After tumor-induced expansion, they can be divided in two main subsets, depending primarily on their ancestors, but also on the suppression mechanisms they exert: monocytic MDSCs, with a CD11b^+^LY6G^−^LY6C^high^ phenotype, and granulocytic MDSCs, with a CD11b^+^LY6G^+^LY6C^low^ phenotype. In humans, MDSCs are characterized as CD11b^+^CD14^−^CD33^+^ cells [46] and have been found to be elevated in patients with different types of cancers [42, 47, 48]. As indicated by their heterogenic composition, MDSCs can inactivate both CD4^+^ and CD8^+^ T cells [49–52] and therefore display a large array of mechanisms of T cell function suppression. One such mechanism is represented by tumor-induced overexpression of CD80 (B7-1) on the surface of MDSCs, to which the inhibitory CTLA-4 (CD152) molecule expressed on CD4^+^CD25^+^ T cells binds with high affinity. Binding of CTLA-4 to CD80 was shown to induce suppression of antigen-specific immune responses [53]. High production of arginase [54, 55] constitutes a common suppressive mechanism for all subsets of MDSCs. Granulocytic MDSCs particularly produce high levels of ROS [45], through signaling via the STAT3 pathway, thus leading to direct damage of the T cell DNA. In contrast, monocytic MDSCs present increased iNOS activity leading to high NO production [56]. In their turn, increased levels of NO have the capacity to induce T cell function suppression via different inhibition mechanisms of MHC class II expression [57] or STAT5 signaling cascade [58].

Other mechanisms of MDSC-induced suppression of effector T cells comprise induction of Tregs by IL-10 secretion in mouse models of colon carcinoma, B16 melanoma, and in patients with HPV-induced neoplasia [59]; depletion of cysteine, the essential aminoacid necessary for T cell activation [60]; secretion of high peroxynitrite levels, which lead to tumor progression [61] upregulation of Cox2/PGE2 [62]. However, the suppressive capacity of MDSCs has been recently questioned by a highly controversial study [63] proving that MDSCs from ascites of ovarian cancer bearing mice were immunostimulatory (they increased CTLs proliferation via CD80 signaling) and adoptive transfer of these MDSCs induced tumor regression. Lastly, immature dendritic cells (iDCs) suppress antitumor immunity by induction of Tregs [64], which in their turn inhibit HPV-specific immunity in patients with (pre)malignant cervical neoplasia [65].

#### 2.2.3. Regulatory T Cells (Tregs)

In terms of adaptive immunity, one of the most studied immunosuppressive cell populations is represented by CD4^+^CD25^+^FoxP3^+^ Tregs [66–68]. Based on their phenotype and localization, Tregs can be divided into several categories: naturally occurring FoxP3^+^ Tregs, generated in the thymus [69–72], and antigen-induced Tregs, generated in the periphery [69]. One of the main definitory characteristics of CD4^+^CD25^+^ Tregs of normal naïve mice is represented by the high expression of the TNF-receptor superfamily member GITR (glucocorticoid-induced TNFR-related protein) [73]. A more detailed subphenotypic classification of Tregs can be found in the review of Feuerer et al. [74]. Tregs can suppress the antitumor immune responses through their high surface expression of CTLA-4, the main T-cell inhibitory signal [75] which mediates attenuation of intercellular association. Moreover, FoxP3^+^ naturally occurring Tregs (nTregs) are well-known negative regulators of antitumor immunity through different mediators, such as FoxP3 [76]. Intratumoral accumulation of FoxP3 leads to poor prognosis of gastric [77] and ovarian [78] carcinomas. Another mediator of the antitumor effects of nTregs is IL-2, needed for *in vivo/in vitro* functional Treg activation [79] and maintenance of their CD25 expression [80]. After IL-2 and TGF-*β* stimulation [81], antigen-induced FoxP3^+^ Tregs [82] have also been shown to present suppressive activity.

Th17 T cells represent a proinflammatory subset of helper T cells, particularly characterized by their capacity to secrete IL-17 *ex vivo* and to constitutively express the lineage-specific factor RORyt [83]. Recent studies indicate towards a close relationship between these Th17 T cells and a distinct subset of suppressive human memory CD4^+^FoxP3^+^ Tregs [84]. IL23-induced Th17 cells [85] produce IL17, a cytokine that enhances inflammation by stimulating the expression of other pro-inflammatory cytokines and acute phase proteins. Although there are some studies which indicate an antitumoral function of Th17 cells [86], *in vitro* experiments establishing the pro- or antitumor role of these cells are equivocal. Also, secretion of IL-17 by Th17 T cells promotes neovascularization and tumor growth in a mouse model of ovarian cancer and in patients with advanced cancer [87]. Therefore, additional studies are necessary to clarify the functions of Th17 cells with regards to tumor immunity.

### 2.3. Other Mechanisms of Tumor Progression

The vast majority of mechanisms of tumor Immunosuppression are generated by a complex interplay of activities and factors belonging to effector-extrinsic suppressor cell populations. One such effector-extrinsic mechanism that has been shown to contribute to tumor progression involves the overexpression of some G-protein-coupled receptors (GPCRs) on the surface of endothelial cells. In some cases, the effect of this overexpression was correlated with tumor progression and metastasis.

In contrast to effector-extrinsic mechanisms of tumor development, effector-intrinsic mechanisms evolve based on the upregulation of coinhibitory receptors able to induce direct lymphocyte inactivation. Both the roles of these GPCRs and those of upregulated inhibitory factors on the surface of various immune-cell populations constitute mechanisms contributing to tumor development.

#### 2.3.1. Endothelin Receptors

Endothelin receptor type A (ET_A_R) and type B (ET_B_R) are GPCRs that belong to the endothelin system. For a more extensive review, see Bagnato and Rosanò [88]. Endothelins, the corresponding ligands of the endothelin receptors, are produced in a wide variety of cells, ranging from endothelial to smooth-muscle cells. Synthesis and secretion of endothelin-1 (ET-1), the corresponding ligand of ET_A_R, in these cells can be induced by a large array of stimuli within minutes. ET-1 is not stored in the secretory granules of the endothelial cells [89]; therefore, its production translates to high ET-1 plasma levels. Upon binding of its correspondent ligand ET1 located in the plasma, ET_A_R promotes vasoconstriction and tumor cell proliferation through a phospholipase C dependent mechanism [90]. On the other hand, ET_B_R was shown to regulate T cell adhesion and tumor homing via NO and ICAM-1 [91]. Whether these actions are mediated by ET_B_R interaction with ET-1 or one of the other endothelin ligands still remains to be unraveled. In the context of tumor immunology, expression of ET_A_R has been reported in prostate cancer patients with bone metastasis [92] and HPV-induced neoplasia [93, 94], whereas ET_B_R expression was associated with the absence of tumor infiltrating lymphocytes and decreased survival time of ovarian cancer patients [91]. Also, upregulation of ET_B_R in patients with vulvar squamous cell carcinoma has been correlated with tumor progression and early metastasis [95].

#### 2.3.2. Negative Regulatory Factors

Programmed death-1 (PD-1), a member of the CD28 superfamily of T cell regulators [96], is not only a negative regulator of antitumor immunity, but exhibits a broader expression and function, since PD-1 knock-out mice have been shown to develop glomerulonephritis [97] and cardiomyopathy [98]. Expression of PD-1 can be transiently upregulated on the surface of activated CD4^+^ and CD8^+^ T cells, B and NKT cells and DCs [99]. Also, high levels of PD-1 have been found on chronically activated CD8^+^ T cells and during chronic infections [100]. PD-1 has two corresponding ligands, PD-L1 and PD-L2, members of the B7 family [101]. Within the context of tumor immunology the ligand PD-L1, which presents an almost ubiquitous expression profile, is most relevant. Coinhibitory signaling via PD-L1 (but not PD-L2) is necessary for conversion of naïve CD4^+^ T cells to adaptive CD4^+^FoxP3^+^ Tregs. The PD-1/PD-L1 signaling pathway is viewed as yet another immune escape mechanism of solid tumors [102], due to its capacity to inhibit T cell activation [103] through various downstream signaling effects. Although not as disputed as the PD-1/PD-L1 system, the lymphocyte-activation gene (LAG-3), member of the immunoglobulin superfamily and expressed on the surface of activated regulatory CD4^+^ and CD8^+^ T cells, B cells and NKT cells have also been shown to contribute to tumor immunesuppression, as Tregs from LAG-3^(−/−)^ mice present reduced regulatory activity [104].

#### 2.3.3. Secondary Contributive Mechanisms

Other contributive mechanisms of tumor development involve blockade of the granzyme B/perforin pathway by overexpression of the serine protease inhibitor PI-9/SPI-6 [105], modifications in the antigen presentation system [106], developed resistance of tumor cells to apoptosis, and expression of indoleamine 2,3-dioxygenase (IDO) by the tumor or host stromal cells [107].

A large array of various tolerance and antitumor Immunosuppression mechanisms contribute to orchestrating tumor growth and progression. Therefore, effective mono- or polymodality strategies to improve homing and activity of immune effector cells to tumors need to be developed in order for cancer immunotherapy to succeed. A detailed summary of immunotherapeutic strategies developed so far and their corresponding efficiency will be presented in the next section of this review.

## 3. Shifting the Balance: Strategies to Improve Homing and Activity of Immune Effector Cells to Tumors

To counteract the numerous mechanisms of tumor immune evasion, an ever increasing number of strategies aimed at improving both innate and adaptive antitumor immunity has been developed over time. Based on their overall target aim, these strategies can be categorized as those which attempt to increase homing of effector T cells to tumors and those that, directly or indirectly, increase antitumor activity of intratumoral effector T cells, either by overcoming tumor-induced tolerance or by overriding the immune-suppression mechanisms imposed during tumor development. 

### 3.1. Increased Homing

Due to the large variety of escape mechanisms developed by the tumor microenvironment and the tumor itself, proper trafficking of effector T cells into the tumor may not always occur. An impaired trafficking of these effector cells to the tumor site will give rise to a negative regulatory process, leading to tumor development and progression. Thus, strategies to block this process and enhance homing of effector cells to tumors are of crucial importance for fighting tumor progression. The most widely used strategies to increase recruitment of effector T cells to tumors aim at targeting both the intrinsic alterations of the tumor cells and the extrinsic alterations induced at the level of effector cell populations. These encompass local tumor irradiation, blockade of endothelin receptors, and effector CTL antibody-targeting and taxane-based chemotherapy.

#### 3.1.1. Local Tumor Irradiation

Within the clinical setting, local or total body irradiation is frequently used as adjuvant therapy, in association with other therapies such as surgery, hormonal therapy [108], or bone-marrow transplantation. Evidence is accumulating that local tumor irradiation is able to modulate expression of receptors and cytokines by cancer and stroma cells, resulting in tumor microenvironment changes that can be used for increasing the effects of immune therapy [109, 110]. These changes seem to facilitate recruitment of effector T cells to tumors via two distinct mechanisms: first, by promoting vasculature normalization [111] and second, by stimulating overexpression of endothelial adhesion molecules, such as VCAM-1 [112]. More recent studies indicate that irradiation induces chemokines involved in recruitment of effector T cells, thus converting tumors into “inflamed tissue”, susceptible to the effector phase of the antitumor immune response [113]. For example, a recent study performed by Quezada et al. in which polyclonal CD4^+^ and CD8^+^ T cells, harvested from mice previously treated with anti-CTLA-4 and depleted of Tregs, were adoptively transferred into irradiated mice bearing large tumors indicated increased protection against tumor outgrowth [114]. The result seemed to be at least partly due to irradiation-induced overexpression of ICAM and VCAM by the tumor vasculature and increased infiltration of effector T cells to the tumor site. In our hands, local irradiation of TC-1 (HPV transformed) tumor bearing mice with different irradiation dosages induces increased natural infiltration of both CD8^+^ effector T cells and CD8^+^ specific CTLs. Also, combination of local TC-1 tumor irradiation with adoptive transfer of *in vitro* restimulated CFSE-labeled specific CTLs lead to a significantly increased homing of the specific CTLs to the tumor site (Draghiciu O, Walczak M, Nijman HW and Daemen T, unpublished data). 

#### 3.1.2. Endothelin Receptors Blockade

Various studies demonstrate that endothelial cells from a variety of human cancers overexpress the endothelin receptors. Hence, blockade of these receptors seems to be a promising strategy for delaying tumor development or stopping tumor-cell proliferation. In fact, selective ET_A_R blockade by the experimental drug atrasentan has been shown to delay progression of hormone-refractory prostate adenocarcinoma [115] and enhance the effect of paclitaxel/docetaxel used for treatment of prostate cancer [116] in patients. In a mouse model of HPV-induced cervical carcinoma, blockade of ET_A_R caused inhibition of tumor growth [94]. Although it can be hypothesized that the effect of ET_A_R blockade on tumor growth is mediated by an increase in T cell homing to the tumor site, further studies need to be performed to elucidate the underlying mechanisms. On the other hand, in the context of ovarian and also other cancers, overexpression of ET_B_R was associated with the absence of tumor-infiltrating lymphocytes and short patient survival time [117]. Moreover, it was shown that interaction between ET_B_R and its corresponding ligand ET-1 induces downregulation of ICAM expression, an effect rescued by administration of the small molecule inhibitor BQ-788. Neutralization of ET_B_R by administration of the inhibitory peptide BQ-788 suppressed intercellular communication and cell growth in melanoma nude mice [118] and significantly increased T cell homing to tumors *in vivo* [117].

#### 3.1.3. Antibody-Mediated Targeting of Effector CTLs

Monoclonal antibody therapy is a method most commonly used to functionally inactivate or deplete suppressive immune populations such as MDSCs or Tregs (see Section 3.2.1). However, various studies using bispecific monoclonal antibodies suggest that this approach is a useful tool with a larger therapeutic applicancy. Effector CTL targeting aims at overcoming extrinsic tumor-induced tolerance by making use of bispecific monoclonal antibodies. These particular antibodies are directed against antigens expressed by both activated T cells and tumor cells and display potent *in vitro *[119] and *in vivo* [120] effects against tumor cells. In a severe combined immunodeficiency deficient (SCID) mouse model, this potent antitumor activity has been enhanced even further, due to inhibition of apoptosis of antibody-targeted cytotoxic T cells by costimulation with an anti-CD28 monoclonal antibody [121].

#### 3.1.4. Taxane-Based Chemotherapy

Another relevant tool to increase homing of effector T cells to tumors is chemotherapy with mitotic inhibitors, such as taxanes. Originally, taxanes have been reported to induce mitotic inhibition through disruption of microtubule functionality. Other studies demonstrate their capacity to bind to and block the function of antiapoptotic molecules expressed on the surface of tumor cells, like Bcl-2 [122], thus inducing programmed tumor cell death. A recent study performed by Dirkx and colleagues aimed at investigating whether inhibition of angiogenesis could contribute to overcoming tumor escape from immunity. The results of this study clearly indicated that the angiogenesis inhibitor paclitaxel was capable of increasing leukocyte rolling on the tumor wall vessel and thus infiltration of circulating effector T cells to the tumor [123].

### 3.2. Increased Activity

Targeting homing of immune effector cells to the tumor site may not solve the problem of eradicating tumor development, as cells that do effectively home to tumor metastases are often found to be dysfunctional. These findings point towards existence of various immunosuppressive mechanisms acquired by the tumor microenvironment in order to fight immune-induced cancer regression. T cell anergy due to extrinsic suppression by regulatory cell populations, inhibition by ligands such as PDL-1, the action of inhibitory factors such as TGF-*β*, and metabolic dysregulations by enzymes such as indoleamine-2,3-dioxygenase (IDO) have all been implicated in generating this suppressive microenvironment. Effective strategies aimed at increasing activity of T cells that migrate to the tumor site address both inhibition of tolerance and restriction of immunosuppression induced by the tumor microenvironment. For targeting the inhibition of the above described negative regulatory mechanisms, several strategies have been employed over time. Some of the strategies most widely and successfully used in both tumor mouse models and cancer patients will be addressed.

#### 3.2.1. Suppressive Immune Populations: Depletion or Functional Inhibition?

One commonly used mechanism of targeting innate as well as adaptive immunity for increasing antitumor activity of effector T cells is depletion of suppressive immune populations. A less intrusive alternative to immune depletion, widely applied as it has been shown to lead to tumor regression [124], consists of manipulating the immune suppressive functions of MDSCs or Tregs. However, functional inhibition of immune suppressive cells, especially directed towards complex and versatile cell populations such as MDSCs, may not be the most suited approach as it is very likely to lead to development of new inhibitory properties causing *de novo* immunosuppression of the previously restored antitumor immune response. 

Different depletion methods, with specificity for the targeted immune population at hand, have been developed over time. Regarding TAMs, selective depletion is promoted by IL-15/TGF-*α* in human primary colorectal adenocarcinomas [125]. Although TAMs depletion can be achieved by different approaches, such as blockade of TAMs chemoattractant chemokines (e.g., blockade of the chemoattractant CCL-2 with the inhibitor molecule bindarit [126] or vaccination with a legumain-based minigene vaccine [127]), the most efficient depletion method involves the usage of clodronate-liposomes. Clodronate-liposomes are artificial spheres formed by dispersion of phospholipid molecules into an aqueous solution of clodronate bisphosphonate. Intraperitoneal or subcutaneous administration of clodronate liposomes induced efficient depletion (75%–92%) of TAMs in both murine teratocarcinoma and human rhabdomyosarcoma mouse tumor models [128] and in a mouse model of human cervical carcinogenesis, respectively [129]. On the other hand, depletion of MDSCs was achieved either by treatment with tyrosine kinase inhibitors, such as sunitinib [130, 131], which also induced reversal of Treg elevation or by treatment with inhibitors of DNA replication, such as 5-fluorouracil [132] or gemcitabine [133]. Also, nTreg depletion was obtained in animal models by administration of anti-CD25 monoclonal antibodies before inoculation of tumor cells [134]. In line with this approach, it was recently reported that selective depletion of FoxP3^+^ Tregs by using transgenic DEREG (depletion of regulatory T cells) mice, in combination with therapeutic vaccination against melanoma, greatly enhanced the antitumor effect [135]. However, other studies in this direction indicate that this combinatorial approach consisting of Tregs depletion and vaccination cannot be generalized for obtaining potent antitumor effects. Depletion of Tregs by treatment with the novel antifolate receptor 4 antibody did not enhance the immune response induced by SFVeE6,7 immunization in a mouse model of cervical carcinoma [136].

Functional inhibition of immunesuppressive properties of negative regulatory cell populations is yet another approach towards improving antitumor immunity. One very good example in this direction is constituted by functional Treg inhibition. Blockade of the main inhibitory effector T cell signal CTLA-4, highly expressed on the surface of Tregs, by using anti-CTLA-4 monoclonal antibodies, has been shown to neutralize Tregs mediated suppression of effector T cells [137]. In a similar manner, GITR blockade with mono- or polyclonal antibodies also neutralized nTregs mediated suppression *in vitro* [138]. Although these suppressive strategies have been proven efficient, positive approaches have also been successfully employed: stimulation of human nTregs through TLR8 has been shown to reverse the inhibitory functions of these cells, via signaling through the TLR8-MyD88-IRAK4 pathway, thus increasing antitumor immunity [139]. However, taking into account the phenotypical and immunesuppressive heterogeneity of MDSCs, functional inhibition of these cells seems to be a more challenging matter. Initially, the suppressive activity of MDSCs has been correlated with the metabolism of L-arginine, the substrate of both iNOS and arginase-1. Accordingly, administration of cyclooxygenase 2 inhibitors has been shown to block production of prostaglandin E2 and thus induce a signaling cascade leading to downregulation of both arginase-1 and iNOS expression on MDSCs. In its turn, this downregulation was associated with increased efficacy of antitumor immunotherapy [140, 141]. However, nowadays an increasing body of evidence points towards ROS and peroxynitrite production as one of the main mechanisms of MDSC-induced effector T cell inhibition. ROS induces effector T cell anergy by direct damage at the DNA level, whereas peroxynitrite is hypothesised to directly nitrosylate intracellular T cell tyrosine to nitrotyrosine, thereby inducing CD8^+^ T cell unresponsiveness [142]. Inhibition of ROS production has been shown to abrogate the MDSCs suppressive effects *in vitro *[45]. Also, *in vitro* treatment of isolated MDSCs with the anti-inflammatory triterpenoid drug CDDO-Me reduced both ROS and peroxynitrite levels, whereas *in vivo* administration of CDDO-Me to tumor bearing mice lead to a significant decrease in tumor size [124]. The reduced tumor size after CDDO-Me treatment could partly be explained by decreased ROS and peroxynitrite production. 

Other effective methods of MDSCs manipulation towards a better outcome of the antitumor immune response include induction of MDSCs differentiation into myeloid DCs that have lost their suppressive activity, by administration of all-trans retinoic acid [143] and inhibition of MDSCs maturation from precursors, by usage of selective inhibitors of the STAT3 maturation pathway [144].

#### 3.2.2. Blockade of Negative Regulatory Factors

Within the context of tumor development and progression, overexpression of negative regulatory factors, such as PD-1 and LAG-3, has often been correlated with chronically activated and nonfunctional CD8^+^ T cells. Hence, blockade of either of these two factors could be an efficient strategy to induce tumor regression. In this context, PD-1 blockade has been shown to increase the induction of effector T cells in the spleen, prolong T-cell proliferation, and enhance recruitment of effector T cells to tumor sites [145]. In multimodality therapy regimens, PD-1 blockade increased therapeutic efficacy of total body irradiation and DCs transfer therapy [146]. Also, antibody blockade of LAG-3 in two murine models of self- and tumor-tolerance increased the accumulation and effector function of antigen-specific CD8^+^ T cells [147]. Thus, combination of mAb therapy against PD-1 or LAG-3 with vaccination strategies has been recently demonstrated to restore the functions of tolerized antigen-specific CD8^+^ T cells [148].

#### 3.2.3. Blockade of TGF-*β* Induced Signaling

Several approaches have been employed to induce high avidity effector T cells in an attempt to target the inhibition of tumor-induced tolerance. One such approach involves blockade of TGF-*β* induced signaling. In a xenograft mouse model of prostate cancer, transfer of tumor-reactive TGF-*β*-insensitive CD8^+^ T cells leads to a 50% decrease in average tumor weight, when compared with tumors of mice which underwent transfer of naïve CD8^+^ T cells [149]. Another approach aimed at manipulating TGF-*β* to improve antitumor immune responses involves generation of TGF-*β* insensitive DC vaccines. Transduced DCs, which have been rendered insensitive to TGF-*β*, maintain their normal phenotype, present upregulated expression of surface costimulatory molecules (CD80/CD86) and induce potent tumor-specific cytotoxic T lymphocyte responses *in vivo *[150].

#### 3.2.4. Blockade of Indoleamine 2,3-Dioxygenase (IDO)

IDO is an enzyme constitutively expressed by both various tumor cell lines and diverse human tumors, such as cervical, pancreatic, and colorectal carcinomas. IDO-expressing tumors were shown to block antigen-specific T cell proliferation, thus mediating the process of tumor immune escape [151]. Plasmacytoid dendritic cells (pDCs) resident in tumor-draining lymph nodes were also shown to express high levels of functionally active IDO, which mediated suppression of reactive T lymphocytes. Since IDO catalyzes the first step in the metabolism of tryptophan, IDO activity in tumors and pDCs altogether can be inhibited by various tryptophan analogues such as 1 methyl-tryptophan. Inhibition of IDO activity in pDCs by administration of 1 methyl-tryptophan leads to reversal of T cell suppression [152].

#### 3.2.5. CTLs Manipulation Strategies

So far, proper targeting and usage of different methods to increase host antitumor immune responses have been discussed. However, one other option to consider when aiming for tumor eradication consists of developing strategies which target the expansion and activation of the effector cell populations themselves. Within this context, adoptive cell therapy or other direct CTL manipulation strategies (such as cisplatin treatment) might just do the job.

**(a) Adoptive Cell Therapy (ACT)**

Adoptive T cell therapy is a very widely used clinical method employed for cancer treatment. More extensive reviews regarding ACT have been written over time [153–155]; here, some aspects of ACT relevant to the induction of tumor regression will be briefly highlighted. T-cell-based ACT constitutes another approach of increasing effector T-cell number and activity, by *in vitro* expansion of a patient's own CTLs and *in vivo* reinfusion of the expanded CTLs into the patient himself, associated or not with concomitant exogenous administration of IL-2. ACT can be viewed as a method of indirectly manipulating the immune system towards the induction of a new CTL population. In many cases, lymphodepletion is required before CTL reinfusion, in order to eliminate Tregs or other competing own lymphocytes. Several studies have already been performed in this direction and the highest efficiency of ACT has been reached in patients with metastatic melanoma [156]. In the clinical setting, ACT has been used either as mono- (e.g., expansion and conversion of Tregs [157]) or in polymodality treatments, in combination with gene therapy [158] or total body irradiation to achieve lymphodepletion [159–161].

**(b) Platinum-Based Chemotherapy**

Originally, it was shown that platinum-based chemotherapy leads to tumor cell apoptosis by binding to and causing DNA crosslinking. Recent studies aiming to completely unravel the effects of platinum-based chemotherapy on CD8^+^ T cell mediated immunity reported that cisplatin greatly enhances E7-specific CD8^+^ T cell immunity induced by DNA vaccination in TC-1 tumor bearing mice [162]. Also, combined chemotherapy treatments with the two platinum-based drugs paclitaxel and carboplatin resulted in improved survival in advanced ovarian cancer patients [163]. The observed improved survival might be explained by a synergistic effect of combined therapy, leading to induction of higher cytotoxic T lymphocytes frequencies. However, platinum-based drugs do not constitute the only chemotherapeutics capable of enhancing the antitumor immune function of effector T cells. The alkylating drug cyclophosphamide (CTX) is yet another agent widely used in the chemoimmunotherapy of tumors. CTX has been shown to synergize with exosome-based vaccines by abolishing the Tregs suppressive function and enhancing the vaccine-induced CTL responses in murine tumor models [164]. Also, CTX treatment has been shown to induce differentiation of CD4^+^ Th17 cells in cancer patients [165].

#### 3.2.6. Therapeutic Vaccination

Although the mechanisms by which chronic viruses or bacteria infections promote cancer are quite diverse, a common feature is given by the fact that development of cancer takes place in the setting of chronic infections [166]. To this end, prophylactic immunization strategies have been developed to reduce cancer burden and a very suitable example is given by production of the two prophylactic vaccines Gardasil and Cervarix for HPV-induced cervical cancer. Regarding therapeutic vaccination, Provenge, containing the active substance sipuleucel-T, is the first therapeutic cancer vaccine that demonstrated effectivenessby prolonging life of patients with metastatic prostate cancer [167]. Sipuleucel-T is constituted by peripheral blood mononuclear cells activated *ex vivo* by the recombinant human PAP-GM-CSF fusion protein (prostatic acid phosphatase-granulocyte-macrophage colony-stimulating factor [168]). 

Another therapeutic vaccine, which induced both strong, long-lasting CTL responses in a mouse model of cervical carcinoma and effective eradication of established tumors of HPV-transformed cells [169, 170], is the recombinant Semliki Forest virus vaccine (rSFV). Constituted of a fusion protein of HPV16 E6 and E7 (SFVeE6,7), this vaccine was able to induce specific CTL activity in immune-tolerant, E6/E7 transgenic mice [171]. A comparative study between the prime-boosting efficacy of SFVeE6,7 and that of the recombinant adenovirus type 5 vector expressing the same antigen construct (Ad-eE6,7) revealed that SFVeE6,7 vaccination lead to higher precursor CTL frequencies and activity when compared to Ad-eE6,7 vaccination. The efficacy of SFVeE6,7 vaccination in murine tumor treatment experiments was significantly higher than that of the Ad-eE6,7 counterpart [172]. Also, low doses of IL-12 expressed by a SFV virus vector (SFV-IL12) augmented the antigen-specific and antitumor responses induced by the virus vector alone [173]. More recent studies showed that the rSFV vaccine induces strong CTL responses in both homologous [174] and heterologous [175] prime-boost immunization regimens in a mouse model of cervical cancer. However, contrary to the excellent therapeutic antitumor responses observed in animal tumors, the clinical results in patients are modest. Explanations for this outcome may be either insufficient activation of antigen-specific immune effector cells or development of immune-suppression mechanisms. For this purpose, development of new multimodality strategies in which vaccination therapies are combined with effective antitumor approaches aimed at increasing homing and activity of immune effector cells to tumors is of crucial importance and thus, an important step forward in cancer immunotherapy.

## 4. Concluding Remarks

In the last few decades, major progress has been achieved within the field of cancer immunotherapy. However, despite this progress, the outcomes of clinical trials performed so far are significantly lower than expected. Contrary to the excellent therapeutic antitumor responses observed in animal tumors, the clinical results in patients are modest. Explanations for this outcome may be either insufficient homing and activation of antigen-specific immune effector cells within the tumor or development of immune-suppressive mechanisms, capable of inhibiting their cytolytic activity. Both recent experimental studies and emerging clinical trials indicate towards development of good vaccination strategies, leading to generation of high levels of effector T cells with a proper phenotype and specificity, as a possible answer to the problem. A desirable, highly effective vaccination strategy should accomplish two purposes. On one hand, it should aim at increasing both the recruitment of antigen-specific effector T cells to the tumor site and their intratumoral arrest for the time necessary to exert their antitumor activity. For this purpose, combination of vaccination regimens, leading to induction of high levels of antigen-specific effector T cells, with ways to enhance homing of these cells to the tumor site, such as local tumor irradiation, endothelin B receptor blockade, antibody-mediated targeting of effector CTLs, or taxane-based chemotherapy, could be a promising strategy. On the other hand, targeting only the homing of vaccine-induced effector T cells to the tumor site might not be enough. We may speculate that once these cells have reached the tumor, they can be anergized or tolerized by diverse immune-suppressive mechanisms developed by the tumor itself or by secondary immune-suppressive populations. To counteract this effect, strategies which aim at maintaining or potentiating the activity of these intratumoral antigen-specific effector T cells, such as depletion or functional inhibition of immune-suppressive populations, blockade of negative regulatory factors, CTLs manipulation methods, or therapeutic vaccination are stringently necessary. 

Concluding, development of new multimodality strategies in which vaccination therapies are combined with effective antitumor approaches aimed at increasing homing of immune effector cells to tumors and their intratumoral activity is of crucial importance and might represent the next step forward in cancer immunotherapy.
